# Evaluation of Pictorial Dietary Assessment Tool for Hospitalized Patients with Diabetes: Cost, Accuracy, and User Satisfaction Analysis

**DOI:** 10.3390/nu10010027

**Published:** 2017-12-28

**Authors:** Dwi Budiningsari, Suzana Shahar, Zahara Abdul Manaf, Nor Azlin Mohd Nordin, Susetyowati Susetyowati

**Affiliations:** 1 Dietetic Programme, School of Healthcare Sciences, Faculty of Health Sciences, Universiti Kebangsaan Malaysia, Jalan Raja Muda Abdul Aziz, Kuala Lumpur 50300, Malaysia; budiningsari25@gmail.com (D.B.); zaharamanaf@ukm.edu.my (Z.A.M.); 2 Department of Health Nutrition, Faculty of Medicine, Gadjah Mada University, Farmako Sekip Utara Street, Yogyakarta 55281, Indonesia; susetyowati@ugm.ac.id; 3 Physiotherapy Programme, School of Rehabilitation Sciences, Faculty of Health Sciences, Universiti Kebangsaan Malaysia, Jalan Raja Muda Abdul Aziz, Kuala Lumpur 50300, Malaysia; norazlin8@ukm.edu.my

**Keywords:** energy and protein intake, dietary assessment tool, pictorial tool, cost, satisfaction, hospitalized patients

## Abstract

Although nutritional screening and dietary monitoring in clinical settings are important, studies on related user satisfaction and cost benefit are still lacking. This study aimed to: (1) elucidate the cost of implementing a newly developed dietary monitoring tool, the Pictorial Dietary Assessment Tool (PDAT); and (2) investigate the accuracy of estimation and satisfaction of healthcare staff after the use of the PDAT. A cross-over intervention study was conducted among 132 hospitalized patients with diabetes. Cost and time for the implementation of PDAT in comparison to modified Comstock was estimated using the activity-based costing approach. Accuracy was expressed as the percentages of energy and protein obtained by both methods, which were within 15% and 30%, respectively, of those obtained by the food weighing. Satisfaction of healthcare staff was measured using a standardized questionnaire. Time to complete the food intake recording of patients using PDAT (2.31 ± 0.70 min) was shorter than when modified Comstock (3.53 ± 1.27 min) was used (*p* < 0.001). Overall cost per patient was slightly higher for PDAT (United States Dollar 0.27 ± 0.02) than for modified Comstock (USD 0.26 ± 0.04 (*p* < 0.05)). The accuracy of energy intake estimated by modified Comstock was 10% lower than that of PDAT. There was poorer accuracy of protein intake estimated by modified Comstock (<40%) compared to that estimated by the PDAT (>71%) (*p* < 0.05). Mean user satisfaction of healthcare staff was significantly higher for PDAT than that for modified Comstock (*p* < 0.05). PDAT requires a shorter time to be completed and was rated better than modified Comstock.

## 1. Introduction

Nutritional screening upon the admission of patients to hospitals is obligatory [1,2]. The Ministry of Health of Republic Indonesia has emphasized the importance of nutrition care process for all patients with nutritional risks, and one of these steps is the monitoring of the patients’ food intake [3]. Furthermore, the Indonesian Government has specified that every hospital must meet the minimal standards for hospital nutrition service, including the requirement that patients’ food intake should be not less than 80% of the recommended daily intake [3]. Monitoring patients’ food intake is therefore mandatory. A survey involving 4512 nurses and doctors in Denmark, Norway, and Sweden found that almost 90% of the respondents agreed that monitoring of a patient’s energy intake should be considered an essential part of the ward rounds routine [4]. The accurate collection of dietary intake information for patients is a difficult and resource-intensive task, therefore, a simple and reliable method for tracking food intake under clinical settings is needed [5,6,7]. 

Visual estimation of dietary intake was first reported by Comstock, St. Pierre, and Mackierman (1981) [8] in a study that involved estimation of school meals consumption comparing children’s self-reports and the weighed method by trained observers. Since this report, several studies have conducted some quantitative validation tests of the visual estimation method in various food service settings, including hospitals [9,10,11,12,13,14]. Although there are some validation studies to record food intake in clinical settings, studies reporting the validity of estimating semisolid or amorphous food items are scarce [11,13]. In addition, developing and validating food recording methods that are country-specific, particularly in Asian countries, which have different food textures and characteristics, is essential [15]. The modified Visual Comstock Scale has been a preferred method for estimating patient food waste in Indonesian hospitals; however, this method requires skilled staff, routine training and support, and also tends to overestimate food intake [16]. Such overestimation can allow problems to go unrecognized by nursing staff, thus preventing follow-ups with further nutritional assessment [9,17]. We have successfully developed and validated a dietary assessment tool for use in a clinical setting, the Pictorial Dietary Assessment Tool (PDAT) [18]. This method is a simple, easy-to-use, quick tool that enables staff to estimate dietary intake and can easily be integrated into dietitians’ evaluations of patients during their stay in the hospital. PDAT provides a ready reckoner of energy and protein content of meals that facilitates the nutrient intake estimation without the need for detailed mathematical computation. There were no differences in macronutrient intake estimation across different backgrounds of healthcare staff [18].

Delivering high-quality nutritional care for patients at risk of malnutrition is essential, and its effects on clinical outcomes and costs savings have been well documented [19]. Studies are still lacking on the amount of money that could be saved if appropriate nutritional care and support are implemented [20]. Thus, this study aimed to elucidate the cost of implementing PDAT among hospitalized adult patients with diabetes. Nutritional care for diabetic patients is very important in preventing complications associated with diabetes, especially the management of metabolic control and optimal weight [21]. Dietary monitoring is crucial for diabetes treatment, for both the improvement of current food intake and for aiding in the explanation of any disturbance in metabolic control [22]. Monitoring of food intake requires the accuracy of the dietary reports of diabetic patients. Patients with diabetes who cannot recognize the amount of caloric intake eventually have a poorly controlled status of diabetes mellitus [23]. However, as yet, little effort has been conducted to determine the best food intake monitoring method among patients with diabetes. Thus, in this study, diabetic patients were selected as a sample to determine the cost effectiveness of the newly developed tool as compared to the conventional one. Further, this approach was taken to ensure consistency and homogenety in the patient demographics of which clinical outcomes were also collected. 

In addition, we investigated the accuracy and satisfaction of healthcare staff after implementation of the PDAT in estimating the dietary intake of hospitalized adult patients, in comparison to the usual tool, i.e., modified Comstock.

## 2. Method

### 2.1. Study Design

**Figure 1:**
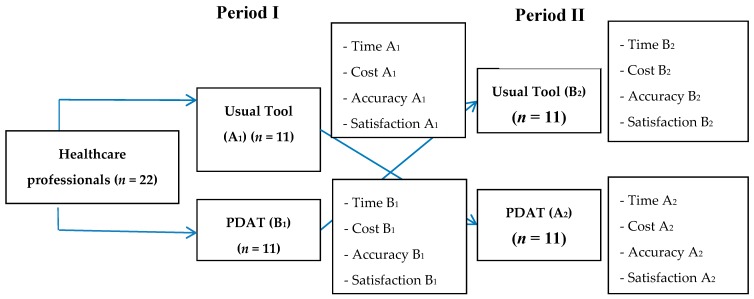
Study Design. Time A_1_ and B_1_: time spent to complete the usual tool by staff group A and B, respectively, in period I. Time A_2_ and B_2_: time spent to complete Pictorial Dietary Assessment Tool (PDAT) by staff group A and B, respectively, in period II. Cost A_1_ and B_1_: cost of implementing usual tool by staff group A and B, respectively, in period I. Cost A_2_ and B_2_: cost of implementing Pictorial Dietary Assessment Tool (PDAT) by staff group A and B, respectively, in period II. Accuracy A_1_ and B_1_: accuracy of usual tool in estimating energy and protein intake of patients by staff group A and B, respectively, in period I. Accuracy A_2_ and B_2_: accuracy of Pictorial Dietary Assessment Tool (PDAT) in estimating energy and protein intake of patients by staff group A and B, respectively, in period II. Satisfaction A_1_ and B_1_: satisfaction using usual tool by staff group A and B, respectively, in period I. Satisfaction A_2_ and B_2_: satisfaction using Pictorial Dietary Assessment Tool (PDAT) by staff group A and B, respectively, in period II.

A cross-over intervention study design was used to address the study purposes. (Figure 1).

### 2.2. Study Setting and Time Scale 

The study was performed in Dr. Sardjito Hospital, a 770-bed tertiary hospital in Yogyakarta, Indonesia, in February and March 2016. A practice trial for all of the healthcare staff as assessors of the study was first conducted in the fourth week of January 2016. 

### 2.3. Ethical Approval

Ethical approval was granted by the Medical and Research Ethics Committee of National University of Malaysia (UKM 1.5.3.5/244/NN-018-2015) and The Medical and Health Research Ethics Committee of Faculty of Medicine Gadjah Mada University-Dr. Sardjito General Hospital, Yogyakarta, Indonesia (KE/FK/351/EC). All respondents were informed about the study and written and signed informed consent was obtained before their inclusion in the study.

### 2.4. Subjects

A total of 132 patients with diabetes were included in this study. Inclusion criteria of subjects were adult patients from a non-intensive care department, diagnosed as having diabetes and not currently going through the procedure of fasting or abstaining from oral food intake. The exclusion criteria were patients receiving only enteral or parenteral nutrition. Patients were included after 24 h of having been admitted to the hospital. Nutrition screening was applied to all of newly admitted patients, using the modified Nutrition Risk Screening-2002 (NRS-2002). Characteristics of the patients consisted of gender, age, the adequacy of energy and protein, diet type, diabetic with or without complication, and the status of nutritional screening (nutritional risk or non-nutritional risk).

### 2.5. Assessors 

The total population of dietitians working in the wards who met the inclusion criteria and worked in the wards of adult patients were invited to participate. Nurses and serving assistants were selected using stratified sampling based on the wards available for hospitalized adult patients, including internal medicine, geriatrics, neurology, and surgery. In this study, healthcare staff needed to fulfil the following criteria: (a) they were employees in the wards as a nurse, dietitian, or service assistant; (b) they had more than three months of work experience in the wards; and (c) they were willing to participate in the study.

As shown in Figure 1, healthcare staff were divided randomly into two groups. In the first period, the first group used the usual tool (modified Comstock) and the second group used PDAT for two weeks. In the second period, the first group used PDAT and the second group used modified Comstock for two weeks. The sample size calculation for comparing means of two groups indicated that a minimum sample size of 10 assessors was required to detect a mean difference in accuracy between the test method and reference method of 13 kcal (given a standard deviation (SD) of the difference between the two value of 10 kcal) [18] with 90% power and a type I error probability of ≤0.05. Each staff member measured three patients for two periods, so that a total of 132 patients were included.

### 2.6. Dietary Intake Measurement Methods

The accuracy of a newly developed dietary monitoring tool, PDAT, to estimate energy and protein intake of patients was compared with the usual tool, i.e., modified Comstock, with a weighing method as a gold standard.

#### 2.6.1. Pictorial Dietary Assessment Tool (PDAT)

**Figure 2:**
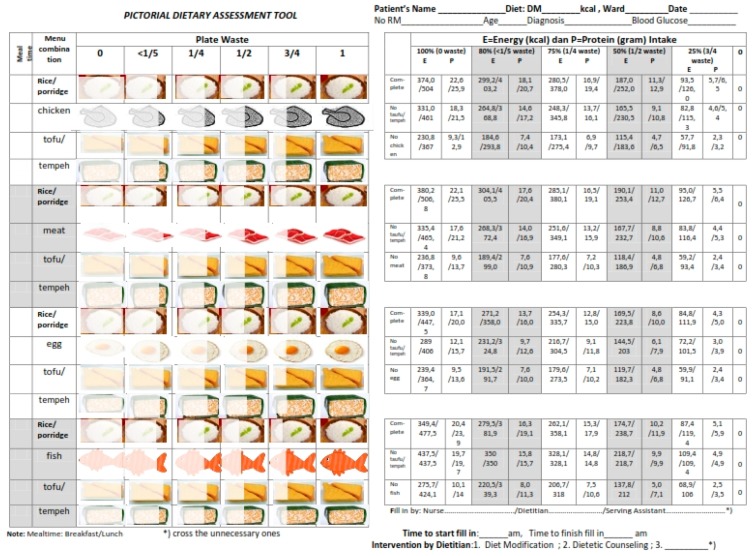
Pictorial Dietary Assessment Tool (PDAT).

PDAT originated from a needs assessment, which was performed by 111 healthcare workers, who consisted of 53 nurses, 27 dietitians, and 31 serving assistants in six hospitals [15], as well as a literature review. The development of PDAT was previously described in detail [18]. With the modification of the modified Comstock six-point scale into the tool, its function is to estimate the proportion of the remaining food left by the patients, including additional information on the meals’ energy and protein content. The PDAT tool is written as a ready reckoner, to enable observers to perform direct estimation on the nutrient intake of patients at each meal. For each level of intake, specific values are given for energy and protein: 390/513 kcal and 23/25 g if 100% rate of food consumption. The energy and protein contents for soft textured food (lower value) and normal textured food (higher value) were represented using these two values. Six pictures of three food groups: staple food (rice/porridge), animal-source protein (chicken, meat, egg, fish), and non-animal source protein (tofu or tempeh) are illustrated in the PDAT. Each picture is partially-to fully-shaded to represent the consumption level (either 0%, 25%, 50%, 75%, 80%, or 100%). The PDAT consists of four possible combinations of menu items (Figure 2). The observers has to select the pictures that best represent the amount of patient’s plate waste, and further estimate energy and protein values related to each level of consumption.

#### 2.6.2. Food Weighing Method

The food weighing method was used as the gold standard. This method was carried out by weighing the plate waste after the patients had finished their meals, following the estimation of each patient’s single meals (breakfast and lunch) using the PDAT or modified Comstock. An electronic kitchen scale (2 kg capacity, accurate to ±1 g) with automatic calibration was used to weigh all the food items. The weighing of the patients’ plate waste and the estimation of the intakes were performed by different personnel, i.e., the healthcare staff estimated intake using PDAT and the investigator performed the food weighing. The amount of leftovers (each remaining food item on the plate, in grams) were then subtracted from the standard portion provided to each patient. 

### 2.7. Cost Estimation Approach

Activity-based costing (ABC) was applied to estimate the cost and time for implementation of the usual tool and the PDAT. In ABC method, activities that consume resources are identified and translated into costs [24]. As shows in Table 1, for each tool implementation, we first identified the processes involved in the nutrition care of patients. Then, we listed each relevant activity performed by the healthcare staff to complete the food intake estimation procedure. We then calculated the cost of each activity involved in the procedure. Activities were defined as direct and indirect costs incurred to record food intake of patients. Since it was difficult to measure indirect costs (such as the loss or increase of productivity), we considered direct costs only, which were directly associated with the food intake recording activity, such as cost of human resource and equipment. 

Human resource consists of nurses, dietitians, and serving assistants who performed the food intake estimation procedure on the study subjects. Cost related to this resource was estimated by multiplying the staffs’ salary per minute with the time needed to complete the procedure using each tool. Salary per minute for each staff category was obtained by dividing salary per month with 22 working days, after which it was further divided by 420 min (for 7 h per day). The estimated activity costs per minute for the nurses, dietitians, and serving assistants were USD 0.03, USD 0.02, and USD 0.01, respectively.

The equipment used in the food intake estimation procedure using PDAT included one set of PDAT forms and stationary. While for modified Comstock, the equipment included one set of modified Comstock forms and stationary. Cost of equipment was estimated by using the current price of the equipment during the study implementation. A proforma was used to document all cost items for both procedures for every subject. 

### 2.8. User Satisfaction Assessment

A questionnaire was created using a 5-point Likert Scale (5 = strongly/totally agree, 4 = somewhat agree, 3 = neither agree nor disagree, 2 = somewhat disagree, 1 = strongly/totally disagree) to determine the satisfaction level of users (nurses, dietitians, and serving assistants) in regards to using the PDAT, in comparison to that using the current estimation tool used in the wards to estimate dietary intake of patients. The questions related to simplicity of usage, user-friendliness, time efficiency, and robustness by means of ensuring its capability of being used by those with minimal training. In order to test face validity and clarity of the questionnaire, ten nurses, dietitians, and serving assistants, were asked to judge whether the questions appeared to be reasonable and cover relevant statements to determine the satisfaction level of users [26]. This resulted in minor linguistic changes. With regard to content validity, the questionnaire displayed good reliability, as the Cronbach’s alpha coefficients (α) ranged from 0.86 to 0.93 for the individual items and 0.89 for the overall user satisfaction rating.

### 2.9. Data Analysis

Descriptive analyses were performed to describe the characteristics of patients. A cost-benefit ratio was calculated by dividing the differences between the mean costs and time in the intervention and control groups by the mean accuracy of dietary intake and staff satisfaction of both groups using an independent *t*-test. Accuracy of estimation of food intake used percentage of estimates within 10% and 15% to compare energy and protein intake using both methods to the “gold standard”—food weighing. 

The total scores of the Likert Scales questionnaire were calculated, including minimum, maximum, mean, median, and standard deviation, and then the results were analyzed by Mann-Whitney test. The level of significance was defined as a *p*-value ≤ 0.05. Statistical analysis was performed using a Windows statistical program package (version 20, SPSS Inc., Chicago, IL, USA).

## 3. Results

### 3.1. Characteristics of Assessors and Patients

The mean age of the healthcare staff was 40 ± 9.3 years and the majority of them were women (77.3%). Additionally, the mean duration of employment among healthcare staff was 19 ± 11.6 years and most of them received higher education, either diploma or bachelor (68.2%) (Table 2). 

There were no differences of characteristics between the two groups of patients measured by PDAT and modified Comstock. The mean age of patients was 56.4 ± 10.5 years and 56.1 ± 10.5 years, respectively, and the majority of patients in both groups were on a 1700 kcal diabetic diet. More than half of the patients (65.9%) were not nutritionally at risk based on screening, and the majority of them had an energy intake below the recommended dietary intake [26] for both breakfast or lunch (62% and 67.4%, respectively). The majority of patients with diabetes were diagnosed with malignancy (29.5%), followed by heart diseases/ischemia (15.2%), renal disorders (14.4%), ulcer (14.4%), cataract (10.6%), and fracture/surgery (6.8%) (Table 3).

### 3.2. Cost Estimation 

The time spent to complete the PDAT form (0.94 ± 0.16 min) was slightly longer than the time for modified Comstock (0.89 ± 0.21 min) (*p* < 0.001). PDAT (1.37 ± 0.61 min) required a shorter time for determining/calculating energy and protein intake than did modified Comstock (2.63 ± 1.12 min) (*p* < 0.001). The total time needed to complete the food intake record of patients by using PDAT (2.31 ± 0.70 min) was, therefore, shorter than that required by using modified Comstock (3.52 ± 1.27 min) (*p* < 0.001). 

The overall time spent to complete food intake recording using PDAT was lower than with modified Comstock by 79 min for the 66 patients. As such, the mean cost for human resources using PDAT (USD 0.05 ± 0.02) was lower than the cost for modified Comstock (USD 0.08 ± 0.04) (*p* < 0.001). A cost saving of as much as USD 0.025/min was, therefore, obtained (Table 4). 

The cost of equipment was slightly higher for PDAT than the cost for modified Comstock (USD 0.22 per patient vs. USD 0.18 per patient, respectively; *p* < 0.001). This difference was caused by the color print cost of the PDAT form, which was more expensive than the modified Comstock form. The total overall cost required for PDAT was also slightly higher than the total cost for modified Comstock (USD 0.27 ± 0.02 vs. USD 0.26 ± 0.04, respectively; *p* = 0.013) after considering the cost for human resource and cost of equipment.

### 3.3. The Accuracy of Nutrient Intake Estimation

The accuracy of the PDAT according to the percentage of estimates within 10% and 15% of the weighed intake (gold standard) was more than 96% for energy intake. The accuracy of modified Comstock was 10% lower than that of PDAT (>86% vs. >96%, respectively; *p* < 0.05), as based on breakfast or lunch meals (Table 5). The accuracy of PDAT in estimating protein intake was significantly higher (>71% of weighed) than that of modified Comstock (<40%). These differences were significant (*p* < 0.05).

### 3.4. Satisfaction of Healthcare Staff

The mean score for the responses to statement “time needed to complete this tool is short enough” was slightly lower for PDAT (4.1 ± 0.35 min) than for modified Comstock (4.4 ± 0.57 min; *p* = 0.004; Table 6). For the statement “it is easy to use (user-friendly) even with minimal training”, the mean score for the responses was also slightly lower for PDAT than for modified Comstock (4.2 ± 0.50 vs. 4.5 ± 0.66, respectively; *p* = 0.003).

In contrast, significantly higher values of mean were obtained for PDAT than for modified Comstock for the responses on other items of the user satisfaction questionnaire (Table 6).

## 4. Discussion

To our knowledge, this is among a very few studies assessing the cost of implementing a newly-developed dietary monitoring tool in a clinical setting, in addition to evaluating accuracy and satisfaction of users. A previous recent study evaluated accuracy, reliability, and efficiency of obtaining energy intake assessment by using the Multi-Component Method for hospitalized patients, without analyzing the costs [28]. The main finding of the current study is that the use of PDAT as a newly-developed visual monitoring tool of patients’ food intake decreased the overall time needed to record patients’ food intake and determine energy and protein intake, in comparison to modified Comstock as the usual tool, and it increased user accuracy and satisfaction at a reasonable cost. 

A recent previous study comparing the use of the Multi-Component Method with the older standard method reported a time saving by 7.3 min [28]. This result was higher than the time saving obtained by the current study (1.2 min), likely because the staff needed more time to choose the appropriate values of energy and protein on the ready reckoner of PDAT, whereas the previous study used the computer program which automatically calculated the nutrients of the food that the patients consumed. Additionally, the previous study did not capture a daily time investment—labor required to download photos and place them in the correct electronic files, and also additional time needed to ensure the computer program remained current with seasonal changes in hospital menus and the addition of new foods [28]. Although the PDAT needs more time to update menus and new foods, this is only conducted once every six months, as the hospital menu will only be revised every six months.

The time saved if the staff used PDAT instead of modified Comstock for 66 patients, which equals 1 h and 19 min, could be devoted by dietitians to perform other relevant activities, such as educating patients regarding their diet and its impact on recovery and health status. Patients with diabetes tend to limit their food intake in order to achieve control of blood glucose; therefore, they are more likely to consume an inadequate diet [29]. Non-value-added activities were also eliminated, such as calculating energy and protein values on a separate sheet, which resulted in more time needed to compute the intake. The modified Comstock estimation as the usual tool has a limitation which was pointed out by previous studies [9]: it requires the nutrient content of foods obtained from a separate nutrient guide in order to calculate the subtotals and total for each nutrient. The time saved by using PDAT in which these non-value added activities were eliminated led to a reduction of human resource costs by as much as USD 1.97 for 66 patients. After considering costs of equipment, the total overall cost for PDAT was USD 0.01 higher than for modified Comstock, due to the color print cost for the PDAT form being more expensive. This cost can be considered low considering the significant increase in accuracy of energy intake that the method yielded. Future efforts to computerize the proforma is needed to reduce the printing cost.

The poor accuracy of protein intake estimation obtained by using modified Comstock may be due to the weakness of this tool as it is applied in hospitals, where data are collected by estimating whole meal waste in general: assessors estimate food waste based on staple foods (rice/porridge) only. This possibility is supported by a study in Japan, which found that estimations from whole trays were less accurate than evaluations based on food items [30]. PDAT allows the estimation of high protein-content dishes from both animal sources (chicken, meat, fish, eggs) and non-animal sources (tofu, tempeh), in addition to staple food. PDAT was developed based on the findings of our earlier needs assessment study [15], that the more desirable dietary assessment tool is not only more practical by reducing time requirements and human resources, but also has good accuracy in estimating patient food intake. Specific food items should, therefore, be prioritized over others for recording on the dietary assessment tool to prevent or treat protein energy malnutrition, such as high protein-content dishes and high energy-content dishes (rice, bread). 

This explanation was supported by the finding that the majority of assessors rated higher satisfaction for the use of PDAT, as it gives more information for decision-making and further nutrition management of patients with diabetes. Ensuring high accuracy of protein intake estimation for patients with diabetes is important in the management of diabetic care, especially among patients with other co-morbidities. For example, diabetic patients with renal disorders must limit protein intake [31], whereas high-protein, low-carbohydrate diets are recommended for the treatment of persons with diabetes mellitus without renal disorders [32,33]. A prospective study which analyzed 21,523 participants recruited between the years 1990 and 2007 found that higher intakes of total and animal protein were both associated with increased risks of Type 2 diabetes, whereas higher plant protein intake tended to be associated with lower risk of type 2 diabetes [34]. The design of PDAT, which differentiates animal protein from plant protein, allows nursing staff to manage higher plant protein levels and lower animal protein levels for better management of patients with Type 2 diabetes.

PDAT possesses one limitation; there is a possibility that staff could under- or over-estimate nutrient values on the ready reckoner. This possibility was reflected in the accuracy of protein estimation being lower than 80%, especially for lunch meals. This limitation could be overcome by providing adequate training to the staff; training is needed considering the lower mean score for the responses to statement “it is easy to use even with minimal training”, that was slightly lower for PDAT than for modified Comstock. This is because modified Comstock as the usual tool has been used for more than ten years, so that the assessors were much more familiar with using modified Comstock. Different results were obtained in a previous study conducted in the United States [28], which found that the Multi-Component Method was able to produce a very detailed calorie count, because it employed a computer program which generated a nutritional intake assessment, in addition to direct observation and a photograph of the food tray taken by a responsible employee. This study also required intense staff training, including a thorough orientation to the computer program, the digital camera, and the observation protocol, as well as multiple mentored practice sessions [28]. The use of technology to enhance accuracy and speed and minimize the costs and inconvenience of assessing diets will be needed in the future [35]. The initial investment in photographic equipment, technological support, and intensive training of personnel, however, still currently inhibits hospitals in developing countries from using this kind of computer-based dietary assessment tool. However, as mentioned earlier, the use of electronic means (e.g., tablets) can be the consideration of further development of PDAT to offset the additional cost of the color printing, compared with the usual tool.

A lack of documentation of nutritional needs is among the reasons for insufficient nutritional support and nutrition care for patients [36]. Nursing staff often do not record patients’ food intake until serious conditions become apparent, such as drastic weight loss over a short period [37]. Optimal nutritional care for malnourished patients who are waiting for treatment or who are recovering from illness is an essential part of total medical care [20]. Using the PDAT, with a small investment, may contribute to improved nutritional care. Furthermore, there is a need to evaluate its effectiveness in improving clinical outcomes.

## 5. Conclusions

In conclusion, the use of PDAT increased the accuracy of estimates of energy and protein intake by as much as 10% and 30%, respectively, and reduced the total time required to complete the food intake record of patients. With the small additional cost of USD 0.01 per patient, energy and protein intake could be more accurately estimated and nutrition intervention more appropriately designed to improve the management of diabetes care.

## Figures and Tables

**Figure 3:**
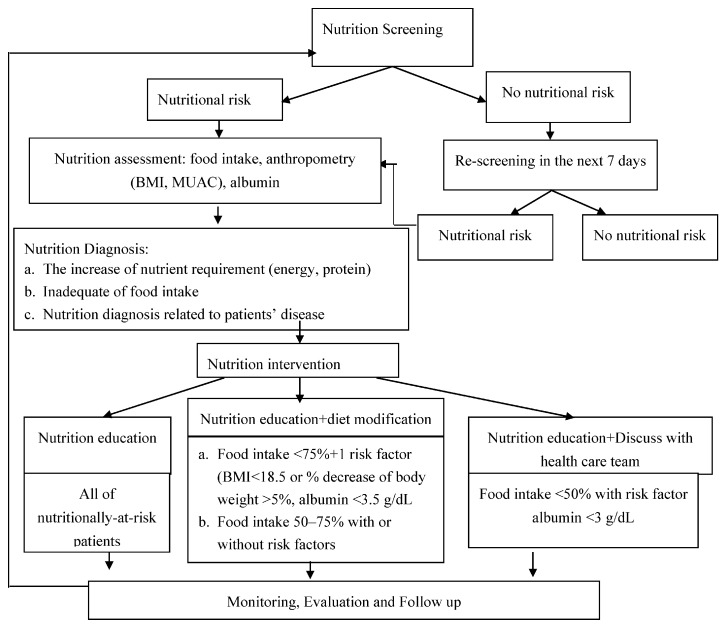
The Nutrition Care Process Algorithm [25]. BMI: Body Mass Index; MUAC: Mid Upper Arm Circumference.

**Table 1:** The development and implementation of the activity-based costing (ABC) method.

Step	Analysis	Description	Elaboration
1	Process Analysis	We described the overall process from the admission of hospitalized patients to the practices to monitor food intake of patients	Refer to Figure 3
2	Activity Analysis	We presented an activity analysis for each relevant activity performed by the healthcare staff to complete the task for food intake recording. It was based on: the process analysis, direct observation, and time measurements. Cost driver was defined as activities consuming cost (labor hour, patient per minute).	dietitians visit patients in order to assign daily diet; nurses record the screening results in order to acknowledge whether patients are at nutritional risk; nurses, dietitians, or serving assistants record food intake of patients; dietitians create a report of patients’ food intake
3	Activity Costs	In order to determine the costs of activities previously identified, we assigned the cost of the resources to the activities using resource drivers. Resources were defined as people, equipment, supplies, etc. that allow activities necessary for the food intake recording of patients.	Cost of resources consist of nurses, dietitians, and serving assistant salaries (USD 267.2/month, USD 229/month, and USD 114.5/month, respectively)
4	Costs of Different Tools to Record Food Intake of Patients	We calculated the cost of the different methods of recording food intake (modified Comstock as the usual tool vs. PDAT).	Refer to The Results Section

**Table 2:** Characteristics of healthcare staff.

Characteristics	Healthcare Staff (*n* = 22)
Age (years), mean ± SD	40 ± 9.3
Gender, *n* (%)	
Women	17 (77.3)
Men	5 (22.7)
Background of Healthcare Staff, *n* (%)	
Nurses	6 (27.2)
Dietitians	8 (36.4)
Serving Assistants	8 (36.4)
Education level, *n* (%)	
Middle (high school)	7 (31.8)
High (diploma, bachelor)	15 (68.2)
Years of working, mean ± SD	19 ± 11.6

**Table 3:** Characteristics of patients according to groups estimated by PDAT and modified Comstock (Presented as mean ± SD and *n* (%)).

	PDAT	Modified Comstock	Total	*p* (Chi Square)
	(*n* = 66)	(*n* = 66)	(*n* = 132)	
Age (years), mean ± SD	56.4 ± 10.5	56.1 ± 10.5		
Gender, *n* (%)				
Women	31 (48.4)	33 (51.6)	64 (48.5)	0.728
Men	35 (51.5)	33 (48.5)	68 (51.5)	
Type of Diet				
Normal textured diet	37 (53.6)	32 (46.4)	69 (52.3)	0.384
Soft textured diet	29 (46)	34 (54)	63 (47.7)	
Diabetic Diet (kcal/day)				
1500	17 (56.7)	13 (43.3)	30 (22.7)	0.836
1700	35 (49.3)	36 (50.7)	71 (53.8)	
1900	11 (45.8)	13 (54.2)	24 (18.2)	
2100	3 (42.9)	4 (57.1)	7 (5.3)	
Adequacy of energy intake (breakfast)				
<RDA ^a^	42 (51.2)	40 (48.8)	82 (62.1)	0.720
≥RDA ^a^	24 (48)	26 (52)	50 (37.9)	
Adequacy of energy intake (lunch)				
<RDA ^a^	44 (49.4)	45 (50.6)	89 (67.4)	0.853
≥RDA ^a^	22 (51.2)	21 (48.8)	43 (32.6)	
Nutrition screening, *n* (%)				
Not at risk	44 (50.6)	43 (49.4)	87 (65.9)	0.854
At risk	22 (48.9)	23 (51.1)	45 (34.1)	
Accompanying diagnosis with diabetes				
Renal disorders	9 (47.4)	10 (52.6)	19 (14.4)	0.259
Hepatic disorders	3 (100)	0 (0)	3 (2.3)	
Malignancy	24 (61.5)	15 (38.5)	39 (29.5)	
Fracture/surgery	4 (44.4)	5 (55.8)	9 (6.8)	
Ulcer	10 (52.6)	9 (47.4)	19 (14.4)	
Coronary heart disease/ischemia	9 (45)	11 (55)	20 (15.2)	
Pulmonary disorders	2 (66.7)	1 (33.3)	3 (2.3)	
Cataract	4 (28.6)	10 (71.4)	14 (10.6)	
Hypoglycemia	1 (50)	1 (50)	2 (1.5)	
Hyperglycemia	0 (0)	1 (100)	1 (0.8)	
Digestive disorders	0 (0)	3 (100)	3 (2.3)	

^a^ RDA-Recommended Dietary Allowance [27].

**Table 4:** Costs to record food intake of patients based on PDAT and modified Comstock.

No.	Resources	Cost Drivers	PDAT	Modified Comstock	*p* Value
	Staff				
1	Number of staff involved	6 nurses	18 patients	18 patients	
		8 dietitians	24 patients	24 patients	
		8 serving assistants	24 patients	24 patients	
		total	66 patients	66 patients	
2	Staff (grade) × salary (USD)	Labor hours (in minutes) mean ± SD	0.02 ± 0.01	0.02 ± 0.01	0.960
		Nurses	0.03/min	0.03/min	
		Dietitians	0.02/min	0.02/min	
		Serving assistants	0.01/min	0.01/min	
3	Time spent by the staff to complete the tool (minutes)	Minute per patient (Mean ± SD)	2.3 ± 0.7	3.5 ± 1.3	0.000 *
4	Staff cost for time spent	(Mean ± SD)	0.05 ± 0.02	0.08 ± 0.04	0.000 *
	Time saved (minute)		1.2		
	Cost saved for the time saved		0.08 − 0.05 = 0.03		
	Time saving gain (total cost/time spent)	USD/minutes	0.03/1.2 = 0.025		
	Equipment				
1	One set of forms	Set per patient	0.07	0.03	
2	Stationary	Set per patient	0.15	0.15	
	Total cost	USD	0.22	0.18	0.000 *
	Total overall cost	(Mean ± SD)	0.27 ± 0.02	0.26 ± 0.04	0.013 *

* *p* < 0.05, Independent t test. 1 USD = IDR 13,100. Cost per minute = cost per month (e.g., for serving assistant: USD 114.5) divided by 22 days, then cost per day divided by 7 h.

**Table 5:** Accuracy of energy and protein intakes estimated by the PDAT and modified Comstock in comparison with food weighing.

	Accuracy	
**Breakfast**	P10 ^a^	P15 ^b^
Energy		
PDAT	98.5	98.5
Modified Comstock	89.4	92.4
Protein		
PDAT	98.5	86.4 *
Modified Comstock	28.8 *	63.6 *
**Lunch**		
Energy		
PDAT	84.8	98.5
Modified Comstock	77.3	93.9 *
Protein		
PDAT	71.2 *	87.9
Modified Comstock	39.4 *	71.2 *

^a^ Percentage of estimates within 10 of food weighing (gold standard); ^b^ Percentage of estimates within 15 of food weighing (gold standard). * *p* < 0.05.

**Table 6:** Satisfaction of healthcare staff towards two methods of dietary assessment of patients.

No.	Satisfaction Aspects	Minimum–Maximum		Median		Mean ± SD		*p* Value
		PDAT	Modified Comstock	PDAT	Modified Comstock	PDAT	Modified Comstock	
1	It is practical enough	4–5	3–5	4	4	4.4 ± 0.49	4.4 ± 0.58	0.477
2	It can be used for all kind of diet (per oral) of patients	2–5	4–5	4	4	4.3 ± 0.69	4.4 ± 0.50	0.403
3	Time needed to complete this tool is short enough	4–5	3–5	4	4	4.1 ± 0.35	4.4 ± 0.57	0.004 *
4	It is easy to use (user-friendly) even with minimal training	3–5	3–5	4	5	4.2 ± 0.50	4.5 ± 0.66	0.003 *
5	It helps for recording food intake of patients	4–5	3–5	5	4	4.6 ± 0.50	4.2 ± 0.72	0.004 *
6	It helps to provide more information on plate waste according to type of food	4–5	2–4	5	2.5	4.7 ± 0.45	2.9 ± 0.96	<0.001 *
7	It helps to obtain the more accurate food intake data	4–5	2–4	5	3	4.7 ± 0.45	2.9 ± 0.88	<0.001 *
8	It facilitates the calculation of energy and protein intake	4–5	2–4	4	2	4.4 ± 0.49	2.7 ± 0.87	<0.001 *
9	It gives more information in decision making for further nutrition management to improve management of diabetic care	4–5	2–5	4	2	4.2 ± 0.39	3.0 ± 0.96	<0.001 *
10	Overall, I would recommend to use PDAT (or Comstock) in other hospitals.	4–5	2–4	4	3	4.2 ± 0.39	3.0 ± 0.80	<0.001 *

* *p* < 0.05 (Mann-Whitney test between PDAT and Modified Comstock).
